# Molecular Screening and Antibiogram Profile of Multidrug‐Resistant Enteropathogenic *Escherichia coli* Isolated From Retail Chicken Meat

**DOI:** 10.1002/vms3.70876

**Published:** 2026-03-09

**Authors:** Sakibul Haque Zilon, Hemayet Hossain, Md. Shahidur Rahman Chowdhury, Sojib Ahmed, Asikur Rahman, Sumaya Shargin Khan, Margia Akter, Bashudeb Paul, Mohammad Ali Zinnah, Monira Noor, Md. Mahfujur Rahman, Md. Masudur Rahman

**Affiliations:** ^1^ Department of Pathology Sylhet Agricultural University Sylhet Bangladesh; ^2^ Department of Veterinary Science and Animal Husbandry Teesta University Rangpur Bangladesh; ^3^ Department of Medicine Sylhet Agricultural University Sylhet Bangladesh; ^4^ Department of Anatomy and Histology Sylhet Agricultural University Sylhet Bangladesh; ^5^ Department of Microbiology and Public Health Gazipur Agricultural University Gazipur Bangladesh

**Keywords:** antimicrobial resistance, chicken meat, *Escherichia coli*, foodborne pathogen, MDR‐EPEC

## Abstract

**Background:**

Enteropathogenic *Escherichia coli* (EPEC) contamination of retail chicken meat is a significant cause of public health concern among susceptible groups like children, the elderly, and immunocompromised individuals.

**Objectives:**

The current research was conducted to identify the prevalence, genotypic pattern and drug resistance of multidrug‐resistant EPEC (MDR‐EPEC) in chicken meat during January to June 2024 in Kuliarchar and Bhairab upazilas, Bangladesh.

**Methods:**

A total of 140 retail chicken meat samples were collected from Kuliarchar and Bhairab upazilas between January and June 2024. The isolates were confirmed by primary culture, biochemical reactions, and molecular confirmation by PCR employing *phoA* and *eaeA* genes. Antimicrobial susceptibility testing was conducted using disk diffusion as per the CLSI 2020 standard.

**Results:**

Overall prevalence of *E. coli* was 68.6% (95% CI: 60.2–76.2) and was found to be higher in Bhairab (81.6%) compared to Kuliarchar (47.2%). 8.6% of these were found to be EPEC. All the retrieved EPEC were resistant to ampicillin, and 91.7% were resistant to ceftazidime and gentamicin. Amoxicillin–clavulanic acid and ciprofloxacin were the most effective antimicrobials. All EPEC isolates (*n* = 12) exhibited resistance to three or more antimicrobial classes and were therefore classified as multidrug‐resistant. Genotypic analysis showed high prevalence of *bla*_TEM‐1, 2_ (91.7%), *bla*_SHV‐1_ (66.7%), *bla*_OXA‐1, 4 & 30_ (33.3%), with *aac(3)‐iv* (83.3%), *sul1* (41.7%) and *tet(A)* (25.0%), indicating widespread β‐lactam and gentamicin resistance in EPEC. The multiple antibiotic resistance (MAR) index was 0.40–0.50, which indicated exposure to high antibiotic pressure.

**Conclusions:**

The findings justify stricter food safety controls, effective use of antibiotics and proper hygiene to provide for the avoidance of the dissemination of MDR‐EPEC through infected chicken meat and public health safety.

## Graphical abstract

Multidrug‐resistant enteropathogenic *Escherichia coli* (MDR‐EPEC) were investigated in retail chicken meat from Bangladesh. Molecular confirmation, antibiogram profiling, and resistance gene detection revealed universal multidrug resistance with high MAR indices, indicating intense antimicrobial pressure and highlighting significant food safety and public health concerns within the poultry production and retail chain.

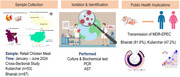

## 1 Introduction

Antimicrobial resistance (AMR) became an international health emergency, threatening public health and food safety severely (Murray et al. 2022). Multidrug‐resistant (MDR) bacterial clones, such as enteropathogenic *Escherichia coli* (EPEC), became more prevalent mainly because of the inappropriate and irresponsible use of antibiotics in human healthcare and animal husbandry and it is a leading diarrheagenic pathotype that causes severe gastrointestinal infections, especially in children and immunocompromised individuals (Anueyiagu et al. 2024; Manyi‐Loh et al. 2018).

EPEC particularly affects infant and young children in low‐ and middle‐income countries (LMICs), where diarrheal diseases remain a leading cause of morbidity and mortality (Anueyiagu et al. 2024). The presence of EPEC in poultry meat highlights its foodborne zoonotic potential. Moreover, resistance determinants carried on plasmids and integrons increase the risk of horizontal gene transfer to human commensals, amplifying the public health burden.

The resistance genes identified in this study, including β‐lactamase and aminoglycoside determinants, are frequently associated with mobile genetic elements such as plasmids, integrons and transposons, facilitating horizontal gene transfer (G. Liu et al. 2022). Upon ingestion of contaminated poultry meat, multidrug‐resistant EPEC (MDR‐EPEC) may transiently colonise the human gut, enabling transfer of resistance genes to commensal microbiota (Z. Liu et al. 2021). This dynamic underscores significant One Health implications linking animal, human, and environmental reservoirs.

One of the main sources of MDR bacteria is the food production chain, and poultry meat is considered to be an important reservoir for antimicrobial‐resistant pathogens (Abreu et al. 2023; Luis Esaú et al. 2019). Poultry farming in Bangladesh cannot be overstated, both in the economy and in the diets; the indiscriminate use of antibiotics in broiler farming has increased the potential of retail‐store chicken meat being contaminated with MDR bacteria, which could lead to zoonotic transmission (S. Chowdhury et al. 2021; M. M. Rahman et al. 2020; Raquib et al. 2022).

High‐level resistance determinants like *bla*_CTX‐M_, *bla*_TEM_, *tetA*, *tetB*, and *mcr‐1* that led to resistance against important antimicrobials used in human medicine were found in retail chicken meat samples when MDR‐EPEC strains were molecularly profiled (Li et al. 2020). The identification of EPEC bacteria resistant to third‐generation cephalosporins that produce extended‐spectrum β‐lactamase (ESBL) on chicken flesh is especially concerning, and this limits therapy and offers a higher likelihood of causing life‐threatening infections in humans (Rahman et al. 2024; Reuland et al. 2013). The transmission of MDR‐EPEC from chicken meat contaminated with the bacteria to humans is facilitated through various mechanisms, including direct contact with uncooked poultry, cross‐contamination during food handling, and eating raw meat (Tadesse et al. 2012; Wardhana et al. 2021). These microbes can infect the human gastrointestinal system after consumption. They can also spread drug resistance genes to commensal flora through horizontal gene transfer factors like integrons, transposons and plasmids (Parvin et al. 2020; Van Boeckel et al. 2019). This kind of gene exchange impairs the effectiveness of infection prevention and therapy by facilitating the persistence and spread of antimicrobial drug resistance in the human microbiome.

The most typical signs of an EPEC infection include fever, nausea, vomiting, stomach pain and watery diarrhoea (Yang et al. 2017). The first identification of diarrheagenic *E. coli* during the infantile diarrhoea outbreaks in the United Kingdom in 1945 was due to the EPEC strains. *E. coli* was detected in chicken in 18 of 64 districts of Bangladesh, with a high overall frequency of 69.3% (M. S. Islam et al. 2023). The prevalence ranged from 24.3% to 100%, which is a significant variation. Furthermore, isolates of *E. coli* showed resistance to 45 distinct antimicrobial drugs from 14 antimicrobial classes, including antibiotics used as a last option and prohibited antimicrobial categories meant to treat illnesses in farm animals. Another study conducted in Bangladesh found that 49.23% of the isolates from broiler chicken and 51.09% of the layer chicken were MDR, and included multiple genes for antibiotic resistance (Rahman et al. 2024).

Globally, the prevalence of MDR *E. coli* has been steadily increasing (Kuenzli et al. 2014). These infections are getting resistant to ever stronger and more recent antimicrobial treatments. Understanding the local antimicrobial sensitivity pattern is crucial for choosing the right antibiotic to treat severe diarrhoea (R. Hoque et al. 2020; Salam et al. 2023). Although several studies from Bangladesh have reported a high burden of antimicrobial‐resistant *E. coli* in poultry (M. S. Islam et al. 2023), pathotype‐specific data on EPEC in retail chicken meat remain scarce, particularly at the upazila (sub‐district) level. Most previous investigations focused on overall *E. coli* prevalence or broiler farm‐level isolates, without molecular confirmation of EPEC (M. S. Islam et al. 2023).

To our knowledge, this is the first study to integrate molecular confirmation of EPEC, resistance gene profiling, phenotypic correlation analysis, and multiple antibiotic resistance index (MARI) evaluation in retail chicken meat at the sub‐district (Kuliarchar and Bhairab upazilas) level in Bangladesh. Therefore, the present study aimed to determine the prevalence, molecular characteristics, AMR patterns, and MARI of MDR‐EPEC isolated from retail chicken meat in Kuliarchar and Bhairab upazilas of Bangladesh.

## 2 Materials and Methods

**FIGURE 1:**
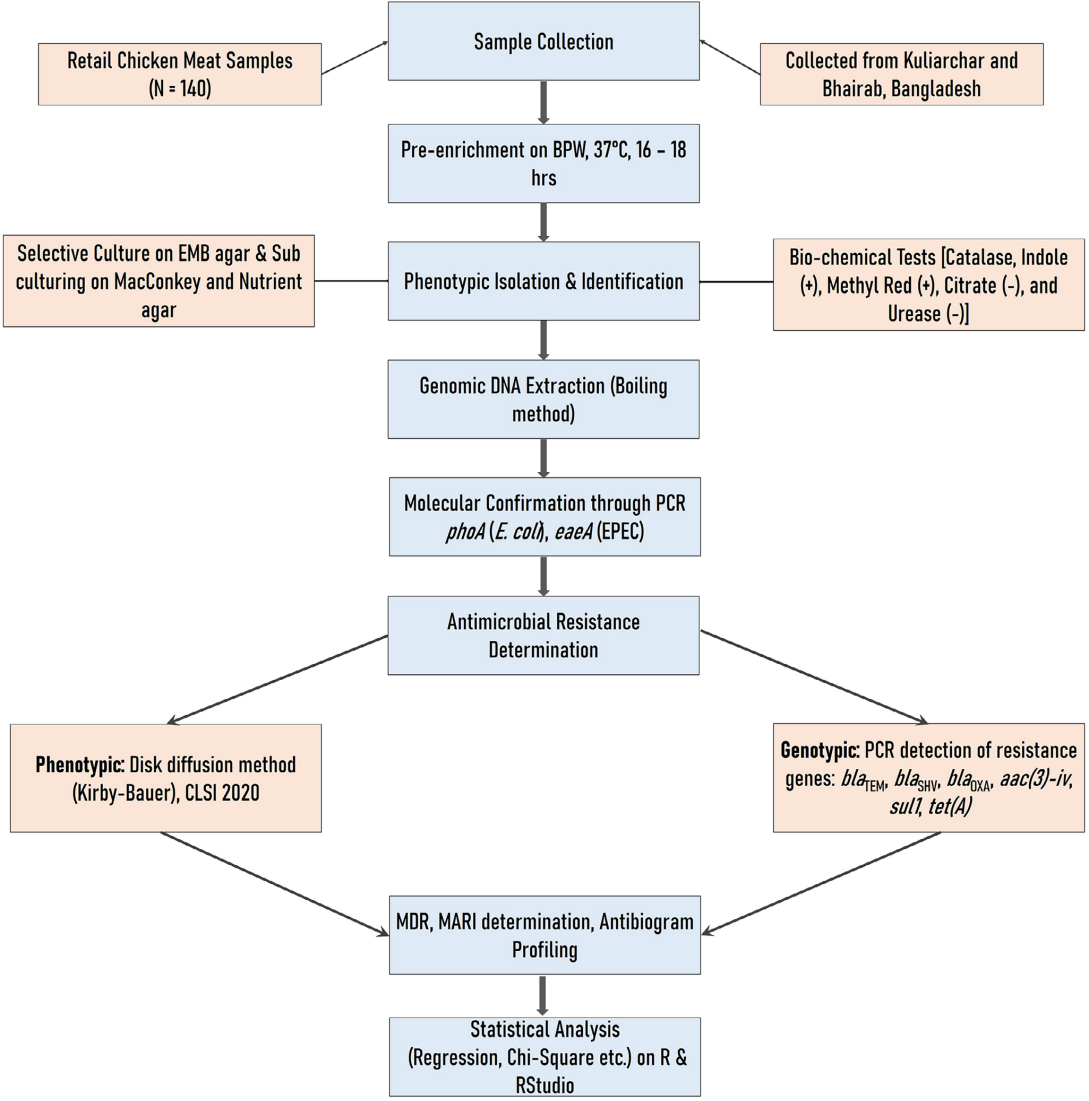
Flow chart illustrating the methodological workflow for isolation, molecular confirmation, antimicrobial susceptibility testing and resistance gene profiling of multidrug‐resistant enteropathogenic *Escherichia coli* (EPEC) from retail chicken meat samples.

A cross‐sectional study was conducted on 140 retail chicken meat samples collected from two upazilas of Bangladesh. *E. coli* and EPEC were identified using culture, biochemical tests, and PCR targeting *phoA* and *eaeA* genes. Antimicrobial susceptibility testing and PCR‐based detection of resistance genes were performed following Clinical and Laboratory Standards Institute (CLSI) guidelines. The detailed methodology is depicted in Figure 1.

### 2.1 Ethical Consideration

The Animal Experimentation and Ethics Committee (AEEC) of Sylhet Agricultural University, Sylhet, gave its approval for this study along with the animal use protocol number (#AUP2022039). This study did not involve any human participants or direct human biological samples, and no procedures were performed that could cause harm to humans. Informed consent from vendors was obtained verbally before sample collection.

### 2.2 Sample Collection and Study Design

At Kuliarchar and Bhairab, in the Kishoreganj district of Bangladesh, a cross‐sectional study was carried out between January and June 2024. A total of 140 retail chicken meat swab samples were collected using a random sampling approach from registered retail chicken meat shops in Kuliarchar and Bhairab upazilas. A list of officially registered retail shops was obtained from the local authority, and shops were randomly selected using a simple random sampling technique.

Inclusion criteria comprised active retail chicken meat shops operating during the study period (January–June 2024) that sold freshly slaughtered, displayed, or dressed chicken meat. Exclusion criteria included unregistered vendors, temporarily closed shops, shops exclusively selling frozen meat, and vendors unwilling to participate.

Samples were collected at two specific time points, morning (8:00–10:00 AM) and evening (4:00–6:00 PM), to evaluate temporal variation in contamination. To assess seasonal variation, sampling was conducted during both the dry season (January–March) and rainy season (April–June). Sampling was performed at regular weekly intervals to minimise temporal bias and ensure representative coverage of different retail conditions, including hygiene status, meat characteristics (skin‐on/skinless), and slaughtering/selling practices. A total of seven independent variables (location, season, chicken type, sampling time, meat characteristics, slaughtering/selling type and hygienic status of retail shop) were evaluated for association with *E. coli* contamination.

**FIGURE 2:**
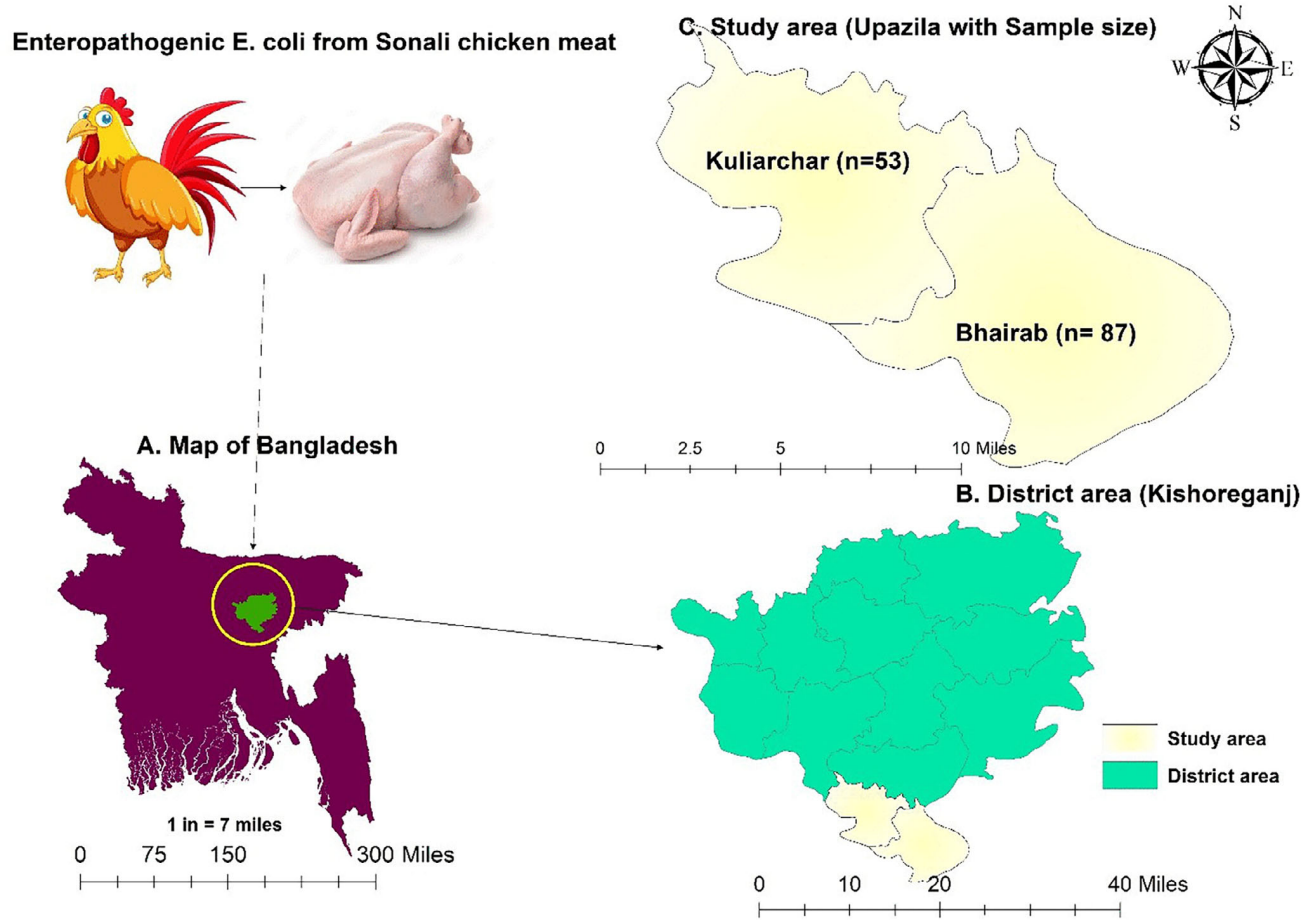
Study area map showing the study area and sample size. The map was created using ArcMap 10.8. (A) Map of Bangladesh, (B) Kishorganj district, (C) selected upazila.

The distribution of the study region, upazila and sample were shown in Figure 2. Swab samples were obtained aseptically using the conventional sampling technique from retail chicken meat stores in the designated sub‐districts. Immediately after obtaining, the swabs were pre‐enriched with buffer peptone water (BPW; Oxoid, UK), kept in a thermal cooler box, and then quickly delivered to the Department of Pathology at Sylhet Agricultural University in Sylhet. After that, the samples were pre‐enriched for 16–18 h at 37°C.

### 2.3 Initial Isolation and Identification of *E. coli*

Following incubation, a sterile loop was used to streak the pre‐enriched samples onto eosin methylene blue (EMB) agar (Oxoid) and incubate them for 18–24 h at 37°C. After subculturing suspected colonies on MacConkey agar (HiMedia Laboratories Pvt. Ltd., Mumbai, India) and incubating them under the same conditions, they were then subcultured on Nutrient agar (HiMedia Laboratories Pvt. Ltd.) for a full day at 37°C (M. S. R. Chowdhury et al. 2025; Rahman et al. 2024). Gram staining was also applied to suspicious colonies. On glass slides, bacterial smears were made, allowed to air dry, and then heated to fix them. After 1 min of crystal violet staining, the slides were washed with water and given a 1‐min iodine solution treatment as a mordant. Following immediate rinsing, decolourisation was carried out using 95% ethanol for 10–15 s. After 30 s of safranin application as a counterstain, the area was cleaned and allowed to air dry. When seen via an oil immersion lens, the stained slides showed *E. coli* as pink, rod‐shaped, Gram‐negative bacteria. Following the methodology by Bhutia et al. (2021), sugar fermentation tests, the methyl red/Voges–Proskauer (MR‐VP) test, citrate utilisation tests, motility tests, indole tests and urease tests were used to further screen presumed positive cultures for biochemical characteristics.

### 2.4 Molecular Detection of *E. coli* and EPEC

With few modifications, the boiling procedure was used to extract the genomic DNA of probable *E. coli* isolates, in accordance with M. F. Hoque et al. (2023). In short, 500 µL of nuclease‐free water was added to a tube containing three to five freshly cultivated colonies. The tube was vortexed and then centrifuged for 5 min at 13,000 rpm. After discarding the supernatant, 500 µL of water devoid of nuclease was added, and the mixture was vortexed. After that, the tube was incubated for 10 min at 100°C in a water bath. Immediately following incubation, it was kept at −20°C for 10 min. The supernatant containing the extracted DNA was then collected and preserved for additional analysis after the tube was spun once more for 5 min at 13,000 rpm.

EPEC was identified using the *eaeA* primer and *E. coli* using the reference primer *phoA* (SFC Probes Ltd., Gyeonggi, Korea) (Table 1). To prepare a 25 µL PCR reaction mixture, we mixed 5 µL DNA sample, 12.5 µL 2× master mix (Add Bio Inc., South Korea), 1 µL each of primer (10 pmol/µL) and 5.5 µL of DNase‐free water. PCR was performed in a thermal cycler following a 5‐min denaturation at 94°C, 30 cycles of denaturation at 94°C for 30 s, 60°C of annealing for 30 s, 72°C of extension for 45 s and a final 10‐min extension at 72°C. The cycler holds the PCR product until it is recovered. One‐minute initial denaturation at 95°C, 35 cycles of denaturation at 94°C for 1 min, 55°C annealing for 90 s, 72°C extension for 90 s, and final extension at 72°C for 10 min comprised the optimal PCR condition for the detection of EPEC. 1.5% agarose gel was made from agarose powder (Addbio Inc., Korea) and 1X TAE buffer. Agarose gel was cooled to 60°C–70°C, added with 5 µL of safe gel dye, and poured with a comb. One hundred base pairs DNA ladder (AddBio Inc.) and PCR products were loaded, electrophoresed for 30 min at 100 V, and observed under UV trans‐illumination.

**TABLE 1:** The primer sequences, amplicon size and target gene for detection of *Escherichia coli* and resistance genes.

Organisms/genes	Target gene	Primer sequence (5′–3′)	Amplicon size (bp)	Annealing temp. (°C)	References
*E. coli*	*PhoA*	F—GGTAACGTTTCTACCGCAGAGTTG R—CAGGGTTGGTACACITGTCATTACG	464	60	Cengiz and Adigüzel (2020)
*EPEC*	*eaeA*	F—AAACAGGTGAAACTGTTGCC R—CTCTGCAGATTAACCTCTGC	454	55	Wu et al. (2010)
*β* lactams genes	*bla*_TEM‐1 & 2_	F—CATTTCCGTGTCGCCCTTATTC R—CGTTCATCCATAGTTGCCTGAC	800	62	Dallenne et al. (2010)
	*bla*_SHV‐1_	F—AGCCGCTTGAGCAAATTAAAC R—ATCCCGCAGATAAATCACCAC	713	62	Dallenne et al. (2010)
	*bla*_OXA‐1, 4 & 30_	F—GGCACCAGATTCAACTTTCAAG R—GACCCCAAGTTTCCTGTAAGTG	564	62	Dallenne et al. (2010)
Antibiotic resistance genes	*tet(A)*	F—GGCGGTCTTCTTCATCATGC R—CGGCAGGCAGAGCAAGTAGA	502	63	M. S. R. Chowdhury et al. (2025)
	*Sul1*	F—CGGCATCGTCAACATAACCT R—TGTGCGGATGAAGTCAGCTC	433	63	Kozak et al. (2009)
	*aac (3)‐iv*	F—AGTTGACCCAGGGCTGTCGC R—GTGTGCTGCTGGTCCACAGC	333	58	M. S. R. Chowdhury et al. (2025)

### 2.5 Molecular Detection of β‐Lactamase and Selected Antibiotic Resistance Genes

PCR amplification of the β‐lactamase genes *bla*_TEM‐1 & 2_, *bla*_SHV‐1_ and *bla*_OXA‐1, 4 & 40_ was performed under standardised conditions following (M. S. R. Chowdhury et al. 2025). The thermal cycling protocol consisted of an initial denaturation at 95°C for 5 min, followed by 30 cycles of denaturation at 94°C for 30 s, annealing at 62°C for 90 s, and extension at 72°C for 1 min, with a final extension at 72°C for 10 min. Each PCR reaction was carried out in a total volume of 25 µL, containing 12.5 µL of 2× PCR master mix (Add Bio Inc.), 0.5 µL each of forward and reverse primers (10 pmol/µL), 5 µL of template DNA, and 6.5 µL of nuclease‐free water.

PCR assays were performed to detect *aac(3)‐iv*, *sul1* and *tet(A)* resistance genes using gene‐specific thermal cycling conditions (M. S. R. Chowdhury et al. 2025). Amplification of the gentamicin resistance gene *aac(3)‐iv* involved an initial denaturation at 94°C for 10 min, followed by 35 cycles of denaturation at 94°C for 60 s, annealing at 63°C for 1 min, and extension at 72°C for 1 min, with a final extension at 72°C for 10 min. For the sulphonamide resistance gene *sul1*, PCR conditions included initial denaturation at 95°C for 15 min, 30 cycles of denaturation at 95°C for 60 s, annealing at 66°C for 1 min, and extension at 72°C for 1 min, followed by a final extension at 72°C for 10 min. Detection of the tetracycline resistance gene *tet(A)* was carried out with an initial denaturation at 94°C for 15 min, 30 cycles of denaturation at 94°C for 1 min, annealing at 63°C for 1 min, and extension at 72°C for 1 min, with a final elongation at 72°C for 10 min.

All PCR reactions were conducted in a total volume of 25 µL containing 12.5 µL of 2× PCR master mix (Add Bio Inc.), gene‐specific forward and reverse primers (0.5–1.0 µL each at 10 pmol/µL), 5 µL of template DNA, and nuclease‐free water to make up the final volume.

### 2.6 Antimicrobial Susceptibility Testing of EPEC

The identified EPEC isolates were tested for antimicrobial susceptibility by the disk diffusion method on Mueller–Hinton agar (MHA) plates (Oxoid), according to CLSI 2020 guidelines (CLSI 2020). The assay was performed using a total of 10 antimicrobials belonging to six different antimicrobial classes (Table 2). The bacterial suspension was uniformly streaked on MHA plates after being calibrated to the 0.5 McFarland turbidity standard. Antibiotic disks were positioned 25 mm apart (centre to centre) after the inoculum had been adsorbed for 5–10 min. The plates were then incubated for 16–18 h at 37°C.

**TABLE 2:** Antimicrobials used in antimicrobial susceptibility testing (AST) in this study.

Antimicrobial class	Name of antibiotics	Code, disk potency	Interpretation		
			Sensitive	Intermediate	Resistant
Penicillins	Ampicillin	AMP 10 µg	≥ 17	14–16	≤ 13
	Amoxicillin/clavulanic acid	AMC 20/10 µg	≥ 18	14–17	≤ 13
Cephalosporins	Ceftriaxone	CTR 30 µg	≥ 23	20–22	≤ 19
	Ceftazidime	CAZ 30 µg	≥ 21	18–20	≤ 17
	Cefpodoxime	CPD 10 µg	≥ 21	18–20	≤ 17
Tetracyclines	Tetracycline	TE 30 µg	≥ 15	12–14	≤ 11
Fluoroquinolones	Ciprofloxacin	CIP 5 µg	≥ 26	22–25	≤ 21
	Levofloxacin	LEV 5 µg	≥ 21	17–20	≤ 16
Sulfonamides	Trimethoprim–sulfamethoxazole	SXT 1.25/23.75 µg	≥ 16	11–15	≤ 10
Aminoglycosides	Gentamicin	GEN 10 µg	≥ 15	13–14	≤ 12

*E. coli* ATCC 25922 was used as the positive quality control strain for antimicrobial susceptibility testing in accordance with CLSI guidelines. For PCR assays, molecular‐grade nuclease‐free water was used as the negative control to monitor potential contamination during amplification.

The diameter of the inhibition zone was then measured in millimetres using a measuring scale. An isolate was termed as MDR if it was resistant to three or more classes of antimicrobials (Liza et al. 2024; M. M. Rahman, Hossain, et al. 2024). According to Naser et al. (2024), the *a*/*b* formula was used to determine MARI, where ‘*a*’ denotes the number of antibiotics to which an isolate is resistant and ‘*b*’ represents the number of antibiotics to which it was tested for resistance. The MARI value greater than 0.2 indicates substantial antibiotic usage in the environment, such as farms, hospitals or contaminated water systems, suggesting that bacteria originating from these sources are likely to exhibit multidrug resistance.

### 2.7 Statistical Analysis

MS Excel was used to perform descriptive statistics. To compare data from various sources, the chi‐square test was performed with a 95% confidence interval (CI). IBM SPSS Statistics version 26 was used for all statistical analyses, and an online sample size calculator (https://sample‐size.net/) was used to compute CIs. To evaluate the associations between the tested antimicrobials, Pearson correlation analysis was carried out using the metan package in R (version 4.3.3) (https://github.com/nepem‐ufsc/metan). Reproducible descriptive tables, univariate and multivariate logistic regression tables, were created using the gtsummary‐R package. Univariable logistic regression analysis was employed to estimate odds ratios (ORs) and corresponding 95% CIs for various sociodemographic variables. To achieve comprehensive adjustment, all biologically plausible factors were incorporated into the multivariable logistic regression model, irrespective of their significance in the univariate analysis. The *p* value was deemed statistically significant if it was less than 0.05.

## 3 Results

### 3.1 Phenotypic Characteristics of Isolated *E. coli*

The phenotypic traits of *E. coli* are circular, opaque colonies with a green metallic sheen on EMB agar. On nutrient agar, the colonies appear large, circular, smooth and moist, exhibiting a creamy white to light yellow colour. The colony appeared round, smooth, and moist with a pink to red colouration on MacConkey agar. The colonies have an entire margin with smooth edges and a raised elevation. *E. coli* looks like Gram‐negative, rod‐shaped bacteria with a pink colouration when stained with Gram stain. Biochemically, *E. coli* was catalase‐positive, coagulase‐negative, citrate‐negative, indole‐positive, methyl red‐positive, urease‐negative and demonstrated positive fermentation for both glucose and lactose in the TSI test. All suspected isolates were confirmed by PCR and subsequently screened for EPEC.

### 3.2 Prevalence of *E. coli* and EPEC

In retail chicken meat samples from the Kishorganj district, the total prevalence of *E. coli* was 68.6% (95% CI: 60.2–76.2). The prevalence of Bhairab was higher (81.6%) than that of Kuliarchar (47.2%) among the study areas (OR = 4.97; 95% CI: 2.35–10.9; *p* < 0.001). The prevalence of EPEC was found 8.6% (95% CI: 4.5–14.5) as shown in Table 3. The univariate chi‐square analysis revealed a statistically significant difference in the prevalence of both *E. coli* and EPEC among retail chicken meat samples (*p* < 0.001), with a significantly higher prevalence of *E. coli* observed in Bhairab compared to Kuliarchar upazila.

**TABLE 3:** Univariate chi‐square test for the prevalence of *Escherichia coli* and EPEC in retail chicken meat samples.

Type	Attributes	*x*/*N*	Prevalence (%)	95% CI	*χ* ^2^ value	*p*
Isolates type					106.36	< 0.001
	*E. coli*	96/140	68.7	60.2–76.2		
	EPEC	12/140	8.6	4.5–14.5		

Abbreviations: *N*, number of samples tested; *x*, number of positive isolates.

### 3.3 Determinants of *E. coli* Contamination

Samples collected during the rainy season (88.5%) showed higher odds compared to the dry season (53.2%) (OR = 6.8; 95% CI: 2.9–18.03; *p* < 0.001). Broiler meat (79.0%) had a higher risk than Sonali chicken (48.1%) (OR = 4.06; *p* = 0.005). Evening samples (83.1%) were more contaminated than morning samples (47.4%) (OR = 5.48; *p* < 0.001). Displayed (86.7%; OR = 5.23) and dressed meat (88.1%; OR = 5.95) showed significantly higher prevalence than freshly slaughtered meat (55.4%). Poor hygienic shop conditions exhibited the highest contamination rate (86.2%; OR = 3.12), indicating increased risk compared with good hygiene practices (Table 4).

**TABLE 4:** Univariate chi‐square test and logistic regression analysis showing the prevalence and associated risk factors of *Escherichia coli* contamination in retail chicken meat samples.

Variables	Prevalence % (*n*/*N*)	Regression analysis			Chi‐square test	
		OR	95% CI	*p*	*χ* ^2^ (df)	*p*
Location						
Kuliarchar	47.2 (25/53)	Ref.			18.12 (1)	< 0.001
Bhairab	81.6 (71/87)	4.97	(2.35–10.9)	<0.001		
Season						
Dry	53.2 (42/79)	Ref.			19.97 (1)	< 0.001
Rainy	88.5 (54/61)	6.8	(2.9–18.03)	<0.001		
Type						
Sonali chicken	48.1 (13/27)	Ref.			8.46 (2)	0.015
Broiler	79.0 (49/62)	4.06	(1.55–10.97)	0.005		
Layer	66.7 (34/51)	2.15	(0.83–5.67)	0.115		
Time of sampling						
Morning	47.4 (27/57)	Ref.			20.06 (1)	< 0.001
Evening	83.1 (69/83)	5.48	(2.57–12.18)	<0.001		
Meat characteristics						
Skinless meat	67.0 (61/91)	Ref.			0.28 (1)	0.593
Skin‐on	71.4 (35/49)	1.23	(0.58–2.68)	0.593		
Meat slaughtering and selling type						
Freshly slaughtered	55.4 (46/83)	Ref.			16.37 (2)	< 0.001
Displayed meat	86.7 (13/15)	5.23	(1.33–34.79)	0.037		
Dressing meat	88.1 (37/42)	5.95	(2.29–18.65)	<0.001		
Hygienic status of the retail shop						
Good	25.0 (3/12)	Ref.			41.55 (2)	< 0.001
Moderate	35.3 (12/34)	0.27	(0.06–1.05)	0.067		
Poor	86.2 (81/94)	3.12	(0.75–11.5)	0.095		

Abbreviations: CI, confidence interval; df, degree of freedom; *n*, number of positive isolates; *N*, number of tested; OR, odds ratio; Ref., reference category.

### 3.4 Antimicrobial Susceptibility Profile of EPEC

**FIGURE 3:**
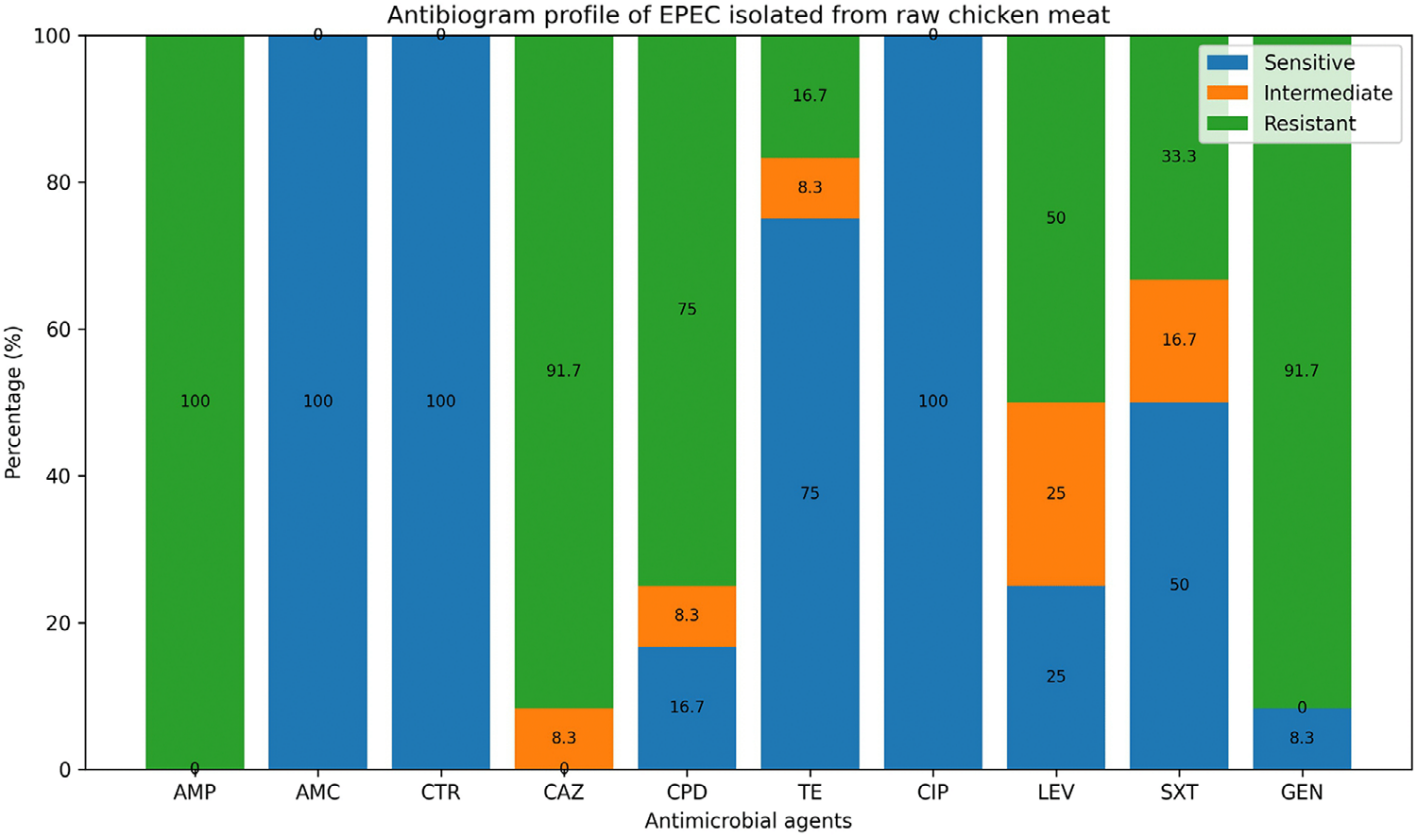
Antimicrobial susceptibility profile of EPEC isolates. AMC, amoxicillin/clavulanic acid; AMP, ampicillin; CAZ, ceftazidime; CIP, ciprofloxacin; CPD, cefpodoxime; CTR, ceftriaxone; GEN, gentamicin; LEV, levofloxacin; SXT, trimethoprim–sulfamethoxazole; TE, tetracycline.

**FIGURE 4:**
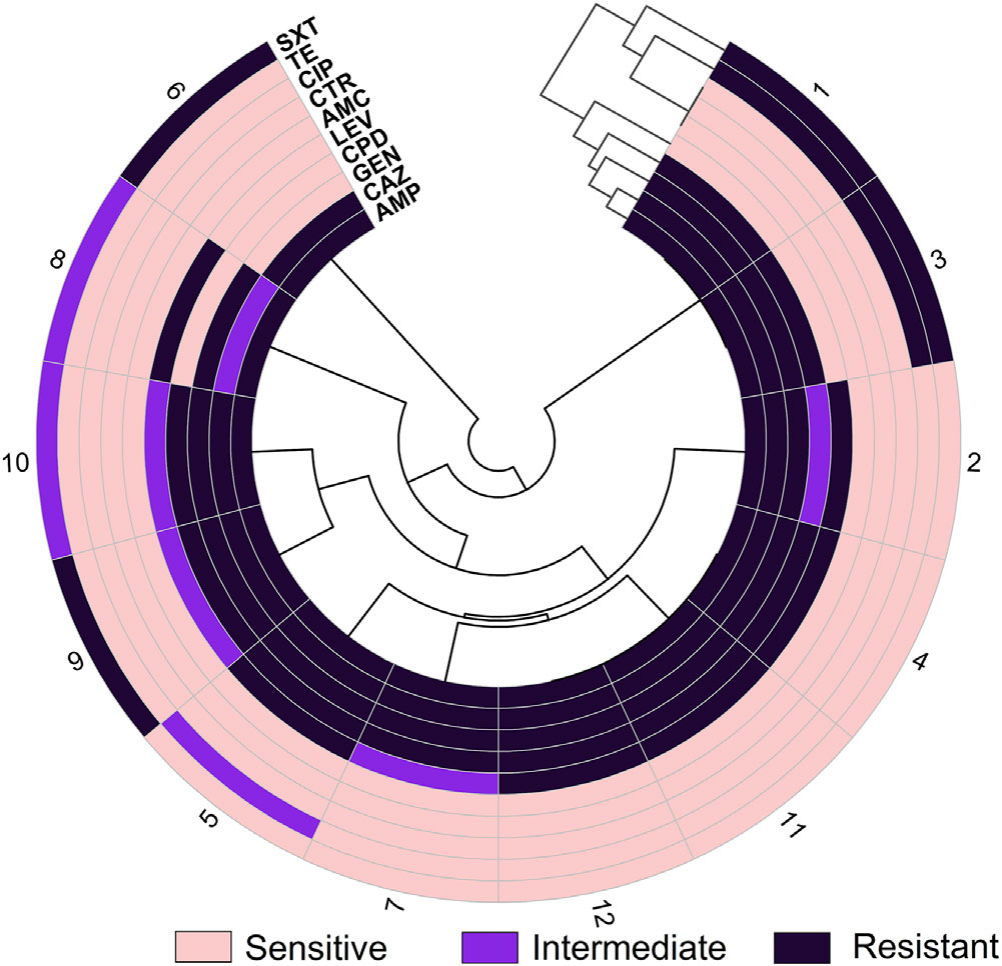
Polar heatmap showing the pattern of resistance of the EPEC isolates.

The Kirby–Bauer disk diffusion technique was used for AST on all samples that tested positive for EPEC. The EPEC isolates from chicken meat revealed varying degrees of resistance against the tested antibiotics (Figures 3 and 4). Notably, all isolates (100%) exhibited resistance to ampicillin, whereas they were completely susceptible to amoxicillin–clavulanic acid and ceftriaxone. There was significant resistance to both gentamicin (91.7%) and ceftazidime (91.7%). Tetracycline demonstrated moderate efficacy, with 75% of isolates being sensitive, while 16.7% exhibited resistance (Figure 3). Ciprofloxacin was the most effective fluoroquinolone, showing 100% sensitivity, whereas levofloxacin had reduced efficacy, with 50% of isolates being resistant. Trimethoprim–sulfamethoxazole also displayed limited effectiveness, with 33.3% of isolates being resistant and 50% sensitive (Figure 3).

### 3.5 MDR Pattern and Phenotypic Correlation

The phenotypic AMR patterns, the number of resistant antibiotic classes, the frequency of each pattern, and the MARI among EPEC isolates from chicken meat are depicted in Table 5.

**TABLE 5:** Phenotypic antimicrobial resistance pattern of Enteropathogenic *Escherichia coli* (EPEC) (*n* = 12) and multiple antibiotic resistance index (MARI) and MDR profile.

Isolates	Phenotypic pattern	No. of resistant antibiotics (class)	No. of phenotypic pattern	Overall MDR %	MAR index
Enteropathogenic *E. coli* (EPEC)	AMP–CAZ–CPD–SXT	04 (3)	01	12/12 (100%)	0.40
	AMP–CAZ–SXT	03 (3)	01		0.30
	AMP–CPD–GEN	03 (3)	01		0.30
	AMP–CAZ–CPD–GEN	04 (3)	05		0.40
	AMP–CAZ–CPD–GEN–TE	05 (4)	02		0.50
	AMP–CAZ–GEN–LEV–SXT	05 (5)	02		0.50

Abbreviations: MAR index, multiple antibiotic resistance index; MDR, multidrug resistant; *x*/*N*, no. of isolates/total no. of isolates tested.

Multidrug resistance or resistance to at least three distinct antibiotic classes, was present in all EPEC isolates (MDR: 100%; 95% CI: 73.53–100.00) (Table 5). Among the 12 isolates, the most prevalent phenotypic resistance pattern was AMP–CAZ–CPD–GEN, observed in five isolates (*n* = 5, MARI 0.40). Other resistance profiles included AMP–CAZ–CPD–GEN–TE (*n* = 2, MARI 0.50) and AMP–CAZ–GEN–LEV–SXT (*n* = 2, MARI 0.50), a very high level of resistance against β‐lactams, aminoglycosides, tetracyclines and sulfonamides were observed.

**FIGURE 5:**
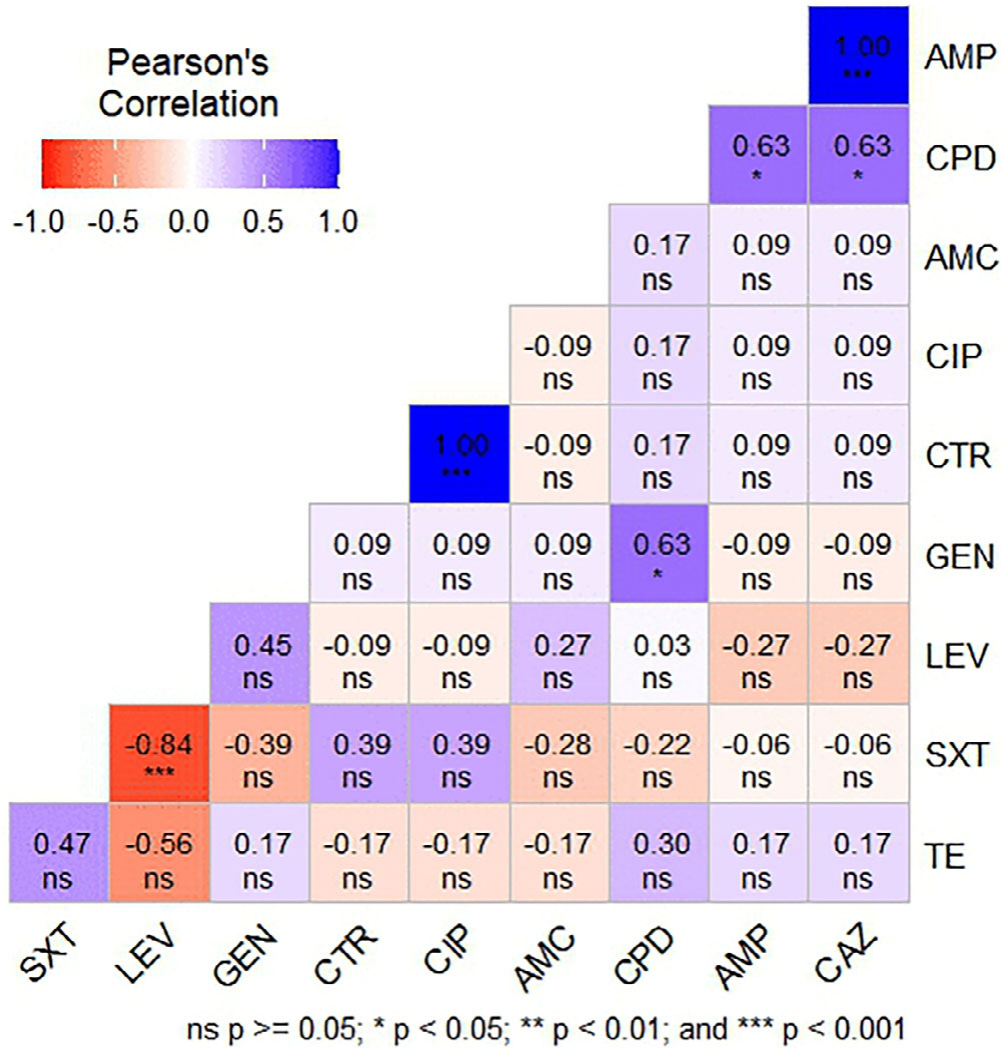
Pearson's correlation coefficient of the antibiotics used in this study. AMC, amoxicillin/clavulanic acid; AMP, ampicillin; CAZ, ceftazidime; CIP, ciprofloxacin; CPD, cefpodoxime; CTR, ceftriaxone; GEN, gentamicin; LEV, levofloxacin; ns, non‐significant; SXT, trimethoprim–sulfamethoxazole; TE, tetracycline.

The correlation heatmap illustrated Pearson's correlation coefficients among AMR profiles of *EPEC* isolates (Figure 5). Both AMP and CPD (*r* = 0.63; *p* < 0.05) and AMP and CAZ (*r* = 1.00; *p* < 0.001) showed strong positive relationships. There was a significant negative correlation between LEV and SXT (*r* = −0.84; *p* < 0.001).

### 3.6 Genotypic Distribution of β‐Lactamase and Other AMR Genes

Genotypic analysis of EPEC isolates revealed a high prevalence of β‐lactamase genes, with *bla*_TEM‐1, 2_ detected in 91.7% of isolates, followed by *bla*_SHV‐1_ (66.7%) and *bla*_OXA‐1, 4 & 30_ (33.3%). Among other resistance determinants, *aac(3)‐iv* was most frequent (83.3%), whereas *sul1* (41.7%) and *tet(A)* (25.0%) were less common (Table 6). Overall, the findings indicate widespread β‐lactam and aminoglycoside (gentamicin) resistance at the genotypic level among EPEC isolates.

**TABLE 6:** Genotypic profiling of β‐lactamase and other antimicrobial resistance genes among enteropathogenic *Escherichia coli* (EPEC) isolates.

Resistance gene type	Genes name	No. of positive	% (95% CI)
β lactams genes	*bla*_TEM‐1 & 2_	11	91.7 (61.5–99.7)
	*bla*_SHV‐1_	8	66.7 (34.9–90.0)
	*bla*_OXA‐1, 4 & 30_	4	33.3 (9.9–65.1)
Antibiotic resistance genes	*tet(A)*	3	25.0 (5.5–57.2)
	*Sul1*	5	41.7 (15.2–72.3)
	*aac (3)‐iv*	10	83.3 (51.6–97.9)

## 4 Discussion

The findings of the present study shed light on the prevalence of MDR‐EPEC on chicken meat available for retail, indicative of the zoonotic potential as well as of the emergent public health concern associated with AMR. The overall prevalence of *E. coli* (68.6%) and EPEC (8.6%) in the retail chicken meat samples of the Kishoreganj district signifies extensive foodborne contamination risks in Bangladesh. The finding is congruent with past studies in Dhaka, Bangladesh, where *E. coli* was recovered in 76.1% of poultry products, to signify the widespread microbial contamination of the poultry chain of Bangladesh (Parvin et al. 2020).

The EPEC prevalence (8.6%) aligns with findings from Bangladesh and India (Pooja Sajish et al. 2025; Parvin et al. 2020), where EPEC prevalence in retail chicken meat typically ranges between 7% and 12% depending on sampling methods and geographic variation. Nepal shows a higher overall pathogenic *E. coli* burden, though specific EPEC percentages vary depending on detection methods (Joshi et al. 2019). Together, these findings reinforce that EPEC contamination in poultry retail meat is a regional public health issue across South Asia, with implications for food safety and AMR.

The significantly higher prevalence in Bhairab (81.6%) compared to Kuliarchar (47.2%) suggests possible regional differences in poultry farming practices, antibiotic usage, and hygiene standards in retail meat markets. Similar findings have been reported in other studies conducted in South Asia, which underscore the urgent need for improved surveillance and regulatory measures to mitigate AMR transmission through the food chain (Van Boeckel et al. 2015). These findings highlight the critical need for a standardised and consistent antibiotic application policy in the poultry industry of Bangladesh (Ibrahim et al. 2023). For instance, the lack of regulation of slaughtering procedures and poor cold‐chain facilities in the developing world are bound to promote bacterial growth (M. A. Islam et al. 2017). The presence of EPEC, a diarrheagenic pathogen, in chicken meat on retail markets depicts its zoonotic capacity that may contribute to slaughtering and manufacturing activities, thus presenting a risk to humans (Mohamed and Habib 2023). This agrees with reports globally of associating poultry‐sourced EPEC with outbreaks of paediatric diarrhoea in resource‐limited areas (Yeda et al. 2024).

Beyond direct zoonotic transmission, poultry‐derived MDR‐EPEC may serve as a reservoir of mobile resistance genes capable of horizontal transfer to human commensal gut bacteria. Resistance determinants carried on plasmids, integrons and transposons can be exchanged within the intestinal microbiome following ingestion of contaminated meat, potentially accelerating community‐level dissemination of AMR through the food chain (G. Liu et al. 2022).

The significantly higher prevalence of *E. coli* and EPEC in Bhairab compared to Kuliarchar may also reflect ecological and anthropogenic differences between the two upazilas. Bhairab is characterised by denser poultry trading networks, higher market throughput, and more intensive handling and storage practices, which can increase opportunities for cross‐contamination and bacterial persistence. In contrast, relatively lower poultry turnover and less centralised retail systems in Kuliarchar may reduce environmental bacterial load. Seasonal humidity, temperature and water quality, particularly during the rainy season, could further enhance bacterial survival and transmission in Bhairab, contributing to the observed regional disparity.

Phenotypic characterisation confirmed the classical features of *E. coli*, including their distinctive colony morphology on selective media, Gram‐negative rod shape and biochemical properties such as indole positivity and lactose fermentation (Erjavec and Erjavec 2023). The confirmation of EPEC through molecular screening further substantiates the pathogenic potential of these isolates, posing a serious risk to consumers. Notably, the high prevalence of MDR among EPEC isolates (100%) is concerning, as it indicates that these pathogens have acquired resistance to multiple classes of antibiotics, making treatment options increasingly limited and the findings are consistent with the findings of another study depicting *E. coli* resistance to three or more antimicrobial drugs (Álvarez‐Fernández et al. 2013). Indiscriminate use, inappropriate selection, inappropriate dose and inappropriate duration of antibiotics at the flock level can be blamed for such a greater prevalence of MDR (Salam et al. 2023). Besides, the high resistance to β‐lactams, particularly ampicillin (100%), is consistent with previous reports indicating the widespread dissemination of ESBL‐producing *E. coli* in poultry (Bhattarai et al. 2024). AMR profile of EPEC isolates also showed significant resistance to ceftazidime (91.7%) and gentamicin (91.7%). These tendencies are due to the extensive use of β‐lactams and aminoglycosides by the poultry sector of Bangladesh, where antibiotics are routinely applied for growth promotion and disease management (S. Chowdhury et al. 2021). Moreover, Fluoroquinolones like ciprofloxacin was 100% sensitive, while levofloxacin was less so with 50% resistance. This variability in susceptibility patterns may be related to variability in fluoroquinolone resistance mechanisms, potentially mediated by *parC* and *gyrA* gene mutations or efflux pump overexpression (Karczmarczyk et al. 2011) The moderate effectiveness of tetracycline, with 75% sensitivity and 16.7% resistance, indicates that even with its extensive history of application in veterinary medicine, the levels of resistance have not yet approached the critical levels seen with β‐lactams and aminoglycosides (Grossman 2016).

The multidrug resistance profile also highlights the heterogeneity of AMR in EPEC. The most common resistance pattern (AMP–CAZ–CPD–SXT), having a MARI of 0.40, indicates widespread exposure to antimicrobials, surpassing the cut‐off value of 0.20, indicating sources of contamination associated with high risk (Ayandele et al. 2020). The strong positive correlation between ampicillin and ceftazidime (*r* = 1.00) and that between ampicillin and cefpodoxime (*r* = 0.63) reflect co‐selection processes, most likely facilitated by plasmids with multiple resistance determinants (M. A. Islam et al. 2017). In contrast, the strong negative correlation between levofloxacin and sulfamethoxazole‐trimethoprim (*r* = ‐0.84) may indicate distinct resistance mechanisms or differing selection pressures in poultry farming. The universal multidrug resistance observed among EPEC isolates suggests sustained exposure to high antimicrobial selective pressure within the poultry production chain. The elevated MARI values (> 0.40) indicate that the source environments are repeatedly exposed to multiple antibiotics, likely due to non‐therapeutic or unregulated antimicrobial use at the farm level. Such selective pressure facilitates the persistence of MDR strains and promotes the retention of resistance determinants on mobile genetic elements, increasing the risk of dissemination along the food chain. The elevated MARI (0.40–0.50) likely reflects sustained selective pressure from non‐therapeutic antibiotic use, including growth promotion and routine prophylaxis in poultry production. In Bangladesh and other LMICs, limited veterinary oversight, poor farm biosecurity, and inadequate compliance with withdrawal periods further facilitate persistence and dissemination of resistant strains (S. Chowdhury et al. 2021; Salam et al. 2023).

The strong positive correlations observed between β‐lactam antibiotics, particularly ampicillin, ceftazidime, and cefpodoxime, may be explained by co‐selection mechanisms mediated by plasmid‐borne β‐lactamase genes, such as *bla*_TEM_ and *bla*_SHV_ (M. S. R. Chowdhury et al. 2025). These genes often coexist within the same resistance cassettes, enabling simultaneous resistance to multiple β‐lactam agents. Conversely, the negative correlation between levofloxacin and trimethoprim–sulfamethoxazole may reflect distinct resistance pathways or differential usage patterns, where selective pressure for one drug class does not necessarily promote resistance to the other.

At the molecular level, the high prevalence of β‐lactamase and aminoglycoside resistance genes is consistent with the observed phenotypic resistance and highlights the role of horizontal gene transfer in shaping the MDR profile of EPEC. The detection of *aac(3)‐iv*, *sul1* and *tet(A)* suggests the involvement of integrons and plasmids in resistance gene dissemination. Collectively, these findings indicate that antimicrobial selection pressure and mobile genetic elements contribute to regional variation and complex resistance patterns in poultry‐associated EPEC.

Whole‐genome sequencing needs to be employed in the future in research to identify resistance genes and assess their risk for zoonotic transmission (Ellington et al. 2017). Bangladesh needs to ban the use of non‐therapeutic antibiotics in poultry more rigorously and educate farmers on alternatives like probiotics and vaccines to prevent the dissemination of MDR‐EPEC (Al Amin et al. 2020). It can also restrict consumer hazards by the use of food safety measures like sanitary slaughter and good chilling, and by increasing consumers' knowledge of safe meat handling (Velazquez‐Meza et al. 2022).

## 5 Limitations and Recommendations

This study was conducted with a limited sample size and restricted to selected sub‐districts, which may limit the generalisability of the findings to the wider district or national level. Although the results provide important region‐specific insights into the burden of MDR‐EPEC in retail chicken meat, they should be interpreted with caution. Future studies should investigate quinolone resistance‐determining regions (QRDR) mutations in *gyrA* and *parC* genes to explain differential fluoroquinolone susceptibility patterns. Future research also incorporating a larger sample size and a more extensive, multi‐regional sampling strategy across Bangladesh would be necessary to substantiate conclusions regarding the national epidemiological situation.

## 6 Conclusion

The high frequency of MDR‐EPEC in Bangladeshi retail chicken meat draws attention to a serious issue with public health and food safety. The results of the study show that multidrug resistance is prevalent, especially to routinely used antibiotics, highlighting the urgent need for strict regulatory actions and antimicrobial stewardship. A great danger of foodborne illnesses is posed by the presence of EPEC in poultry meat, which calls for enhanced hygiene procedures, careful oversight of the use of antibiotics in poultry production, and effective surveillance systems. Campaigns for public awareness and legislative measures are essential to halting the spread of MDR‐EPEC and guaranteeing the consumption of safer food.

To mitigate the public health risk, immediate implementation of enforceable antimicrobial stewardship policies in poultry production is essential. Regulatory authorities should strictly enforce prescription‐only antibiotic sales, prohibit non‐therapeutic and growth‐promoting antibiotic use, and establish a national AMR surveillance program targeting retail meat. Mandatory hygiene certification and routine inspection of slaughterhouses, coupled with structured farmer training on biosecurity and responsible antimicrobial use, are urgently recommended within a coordinated One Health framework.

## Author Contributions

**Sakibul Haque Zilon**: conceptualisation, data curation, formal analysis, investigation, methodology, software, visualisation, writing – original draft, and writing – review and editing. **Hemayet Hossain**: conceptualisation, data curation, formal analysis, investigation, methodology, software, visualisation, writing – original draft, and writing – review and editing. **Md. Shahidur Rahman Chowdhury**: investigation, methodology, software, writing – original draft, and writing – review and editing. **Sojib Ahmed**: investigation, methodology, software, writing – original draft, and writing – review and editing. **Asikur Rahman**: investigation, methodology, software, writing – original draft, and writing – review and editing. **Sumaya Shargin Khan**: investigation, methodology, software, writing – original draft, and writing – review and editing. **Margia Akter**: investigation, methodology, software, writing – original draft, and writing – review and editing. **Bashudeb Paul**: investigation, methodology, software, writing – original draft, and writing – review and editing. **Mohammad Ali Zinnah**: investigation, methodology, software, writing – original draft, and writing – review and editing. **Monira Noor**: investigation, methodology, software, writing – original draft, and writing – review and editing. **Md. Mahfujur Rahman**: conceptualisation, data curation, formal analysis, investigation, methodology, resources, software, validation, visualisation, writing – original draft, and writing – review and editing. **Md. Masudur Rahman**: conceptualisation, data curation, formal analysis, investigation, methodology, project administration, software, supervision, validation, visualisation, writing – original draft, and writing – review and editing.

## Funding

The authors have nothing to report.

## Ethics Statement

The Animal Experimentation and Ethics Committee (AEEC) of Sylhet Agricultural University, Sylhet, gave its approval for this study along with the animal use protocol number #AUP2022039.

## Conflicts of Interest

The authors declare no conflicts of interest.

## Data Availability

The data that support the findings of this study are available from the corresponding author upon reasonable request.
